# Genomic Data Reveals Profound Genetic Structure and Multiple Glacial Refugia in *Lonicera oblata* (Caprifoliaceae), a Threatened Montane Shrub Endemic to North China

**DOI:** 10.3389/fpls.2022.832559

**Published:** 2022-05-09

**Authors:** Xian-Yun Mu, Yuan-Mi Wu, Xue-Li Shen, Ling Tong, Feng-Wei Lei, Xiao-Fei Xia, Yu Ning

**Affiliations:** ^1^Laboratory of Systematic Evolution and Biogeography of Woody Plants, School of Ecology and Nature Conservation, Beijing Forestry University, Beijing, China; ^2^Beijing Museum of Natural History, Beijing, China; ^3^Institute of Wetland Research, Chinese Academy of Forestry, Beijing, China

**Keywords:** conservation units, genetic diversity, *Lonicera oblata*, glacial refugia, Taihang Mountains, threatened species, Yan Mountains

## Abstract

Characterizing genetic diversity and structure and identifying conservation units are both crucial for the conservation and management of threatened species. The development of high-throughput sequencing technology provides exciting opportunities for conservation genetics. Here, we employed the powerful SuperGBS method to identify 33, 758 high-quality single-nucleotide polymorphisms (SNP) from 134 individuals of a critically endangered montane shrub endemic to North China, *Lonicera oblata*. A low level of genetic diversity and a high degree of genetic differentiation among populations were observed based on the SNP data. Both principal component and phylogenetic analyses detected seven clusters, which correspond exactly to the seven geographic populations. Under the optimal *K* = 7, Admixture suggested the combination of the two small and geographically neighboring populations in the Taihang Mountains, Dongling Mountains, and Lijiazhuang, while the division of the big population of Jiankou Great Wall in the Yan Mountains into two clusters. High population genetic diversity and a large number of private alleles were detected in the four large populations, while low diversity and non-private alleles were observed for the remaining three small populations, implying the importance of these large populations as conservation units in priority. Demographic history inference suggested two drastic contractions of population size events that occurred after the Middle Pleistocene Transition and the Last Glacial Maximum, respectively. Combining our previous ecological niche modeling results with the present genomic data, there was a possible presence of glacial refugia in the Taihang and Yan Mountains, North China. This study provides valuable data for the conservation and management of *L. oblata* and broadens the understanding of the high biodiversity in the Taihang and Yan Mountains.

## Introduction

The Earth’s biodiversity consists of approximately 9 million types of organisms (e.g., plants, animals, protists, and fungi), while biodiversity losses have exerted a profound impact on the ecology and society as a whole (Chapin et al., 2000; Cardinale et al., 2012). A total of 10% of tree species (> 8,000) on the earth are threatened with extinction (Cavender et al., 2015). Endemic species are important members of biodiversity hotspots and are of great value to biodiversity conservation (Myers et al., 2000). They are often characterized by narrow geographic ranges and specialized niche requirements, and they tend to have a small population size (Işik and Kani, 2011). Due to the sensitivity to internal factors (e.g., genetic bottleneck, inbreeding depression, and genetic drift) and/or external factors (e.g., human disturbance and environmental randomness), species with small populations are more vulnerable, which may lead to reduced fitness of certain individuals (Charlesworth and Willis, 2009), or even extinction. Moreover, low genetic variation and gene flow are frequently detected in rare plants compared to common plants (Cole, 2003). Understanding the genetic structure of endangered woody species can provide valuable information for their conservation and management.

The Sino-Japanese floristic region holds one of the oldest floras in the North Hemisphere with high species richness (Chen et al., 2018b; Lu et al., 2018). Climatic oscillations and geological events have greatly influenced the genetic pattern and distributional range of many plant species, particularly during the Quaternary glacial–interglacial cycles. Compared to North America and Europe, the fauna and flora in Asia were reported to be less affected by glaciation events. Northern China was less affected by massive ice sheets (Qiu et al., 2011), and an arid belt was suggested to have occurred in this region (Guo et al., 2008). Composing the main part of the Sino-Japanese floristic region, the northern China flora exhibits unique genetic patterns of forestry species. Southern warm-temperate forests mixed with northern cool-temperate forests occur in this region, indicating complicated genetic diversity and genetic differentiation patterns (Ye et al., 2017). It is commonly reported based on limited molecular markers that during the northward expansion of species, there would be a gradual decline in population genetic diversity [e.g., *Acer mono* Maxim. (Guo et al., 2014; Liu et al., 2014) and *Quercus mongolia* Fisch. ex Ledeb. (Zeng et al., 2015)]. In addition, single/multiple refugia in northeast China and high genetic diversity in northern populations are also observed [e.g., *Juglans mandshurica* Maxim. (Bai et al., 2010), *Eleutherococcus senticosus* (Rupr. and Maxim.) Maxim. (Wang et al., 2016), and *Schisandra sinensis* C. Bailley (Ye et al., 2019)]. North China is an important geographic and floristic region of northern China; it contains both numerous rugged mountains and the North China Basin and provides a north–south migration corridor for wildlife. Compared to the substantial progress made by previous literature focusing on the species with a broad geographic range, the genetic patterns of woody plants endemic to North China remain to be clearly understood, especially in the era of population genomics.

Two great mountains are located in North China, namely, the NNE trending Taihang Mountains (THMs) and the EW trending Yan Mountains (YMs), which harbor the highest seed plant diversity in northern China, as well as a high endemism rate (Wang et al., 1995). The THMs lie between the Ordos-Shanxi plateau on the west and the North China Basin on the east, with a north latitude from 34° to 40° (Wang and Li, 2008). The THMs are also a transitional region located between the second staircase (altitude 1,000–2,000 m) and the third staircase (altitude < 500 m) in the three-step topography of China. The intense uplift during the Late Pliocene to Pleistocene (Wu et al., 1999), along with heterogeneous local geographic and environmental changes (Guo et al., 2008; Wang et al., 2018), provided a unique habitat for several species endemic to this region, such as *Opisthopappus taihangensis* (Ling) Shih and *Taihangia rupestris* Yuet Li. The YMs lie in the north of the North China Basin with an east longitude from 115° to 119°. The west end of the YMs is connected to the northeast end of THMs. Most mountains in the THMs and YMs are extremely steep, particularly in the THMs. These two great mountains meet in the northwest of Beijing, and the valley between Changping District on the westside and Yanqing District on the northside are considered as the division. Uncovering the genetic composition of species endemic to the THMs and YMs may help us understand the genetic pattern and preservation of biodiversity in North China and can also allow us to further explore the role of these mountains in the survival and dispersal of endemic species.

High-throughput sequencing (HTS) technology can rapidly detect thousands of single-nucleotide polymorphisms (SNPs) in a cost- and time-efficient manner on a genome-wide scale. Compared to Sanger sequencing method, which generates limited loci, the large data generated from HTS can provide robust phylogeny, reducing the potential incomplete lineage sorting, and enable us to address a wide range of evolutionary questions. Furthermore, a small sample size per population (e.g., a minimum of two individuals) can also generate big data in the population genomic study based on the HTS approach (Nazareno et al., 2017). Thus, it is an effective tool for the assessment of population genetic diversity compared to traditional genetic markers (Glover et al., 2010; Li et al., 2020). Restriction site-associated DNA (RAD) is one of the most effective genotyping-by-sequencing (GBS) methods that allow for extensive SNP discovery on the genome level (Davey et al., 2011; Sonah et al., 2013; Puritz et al., 2014). It has been widely applied in population genetics for numerous model and non-model species (e.g., Wang et al., 2013; Luo et al., 2019; Feng et al., 2020; Xiong et al., 2020; Boukteb et al., 2021; Qiao et al., 2021). In particular, the UGbS-Flex (also denoted as SuperGBS) is a powerful tool for population genetic research with the ability to generate long reads (300–700 bp) and maximize SNP callings irrespective of the species ploidy level, breeding system, and reference genome availability (Qi et al., 2018; Cheng et al., 2020).

**FIGURE 1:**
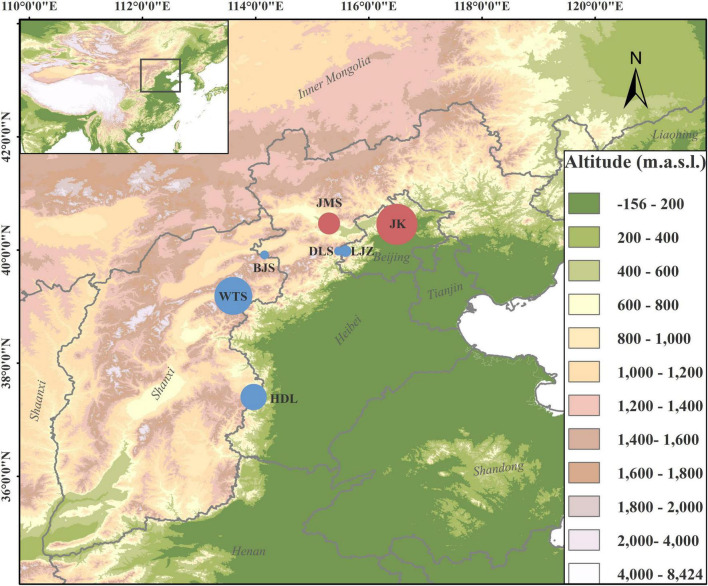
Geographic distributions of sampled *Lonicera oblata* populations. The two red circles on the northside represent populations from Yan Mountains, and the five blue circles represent populations from Taihang Mountains. The circle size corresponds to the population sample size.

*Lonicera oblata* K. S. Hao ex P. S. Hsu and H. J. Wang (Caprifoliaceae) is a critically endangered montane shrub endemic to North China (Zhu et al., 2019; Wu et al., 2021), and it is ranked second in the list of national key protected wild plants in China.^1^ Results from our 13-year field investigation identified seven highly fragmented populations that typically grow in the THMs and YMs (Figure 1). The majority of individuals occurred in habitats of stony and steep cliffs in open forests with an elevation of ca. 1,100 m. Although the red berries of *L. oblata* are frequently observed in the wild, no seedlings are reported. The habitats of several populations are exposed to human activities (e.g., tourism, logging, and grazing), and some individuals are injured directly by cutoff. The highly threatened condition of *L. oblata* may be attributed to the small number and size of populations scattered in such a vast geographic range, unique but extremely fragmented habitats, strong human disturbances, and sensitivity to climate change (Wu et al., 2021). The highly isolated distributional pattern and mountain-top preferred habitat of *L. oblata* is similar to species in the sky islands in southwest China (He and Jiang, 2014), which may suffer both morphological and genetic variation because of local geographic vicariance and population isolation. With the exception of its endangerment status, little is known about the biological and genetic characteristics of *L. oblata*. Thus, research on the genetic diversity and population structure of *L. oblata* at the genome level is imperative for the adequate determination of conservation strategies for policy-makers.

In this study, we employed the novel approach of RAD sequencing, SuperGBS (Qi et al., 2018), on the threatened montane shrub *L. oblata* endemic to the two great mountains, the THMs and the YMs, in North China to (i) assess the population structure and genetic diversity across the full range of the species; (ii) infer the genetic signatures of potential refugia during the Quaternary climate oscillations in the THMs and YMs; and (iii) identify conservation units (CU) and provide suggestions for the conservation of this species. Our results provide both a theoretical basis and a valuable genetic database for the conservation planning of *L. oblata* and contribute to improving our understanding of the preservation and evolution of biodiversity in North China.

## Materials and Methods

### Sample Collection and DNA Extraction

A total of 134 accessions from all seven natural populations covering the entire geographic range of *L. oblata* were sampled. Based on the number of individuals, the seven populations can be classified into four large populations [Heduling (HDL, *N* = 24), Wutai Mountains (WTS, *N* = 34), Jiming Mountains (JMS, *N* = 20), and Jiankou Great Wall (JK, *N* = 36)] located at the species marginal distribution region and three small populations [Bijia Mountains (BJS, *N* = 6), Dongling Mountains (DLS, *N* = 6), and Lijiazhuang (LJZ, *N* = 8)] located at the central region (Figure 1). The only individual found in Beijing Songshan National Nature Reserve, which is near the JK population in Beijing, was not included in this study. Among them, JK and JMS are distributed in the YMs, while the remaining populations are located in the THMs (Figure 1 and Supplementary Table 1). A minimum distance of 30 m from one individual to another was set during the sampling. Leaves were dried in silica gel in the field and stored at −80°C until further use. Voucher specimens were preserved in the herbarium at Beijing Forestry University. Genomic DNA was extracted from the above leaf tissue using the DNAsecure Plant Kit (Tiangen Biotech, Beijing, China) following the manufacturer’s protocol. DNA quantity and quality were determined by Agarose Gel and NanoDrop.

### Preparation of Libraries and Sequencing

Genotyping-by-sequencing was performed following the method described by Qi et al. (2018) using the enzyme combination of *Pst*I/*Msp*I. Briefly, 250 ng of DNA from each sample was double-digested with *Pst*I and *Msp*I, followed by the barcode adapter ligation at the *Pst*I site and a common Y-adapter at the *Msp*I site. Unligated adapters were removed by the recovery system of the improved magnetic bead. Recovered fragments with a length of 300–700 bp were PCR-amplified and tested for density using Qubit2.0 to ensure density values greater than 5 ng/μl. The libraries were subsequently sequenced using the Illumina HiSeq X Ten, PE150 platform at OE Biotech Co., Ltd., Qingdao, China.

### Single-Nucleotide Polymorphisms Calling

Raw reads were split by barcode using Stacks version 2.4 (Catchen et al., 2013) with the option “process_radtags –renz-1 -r -s 0 –retain_header –adapter_mm 1 –adapter_1 –adapter_2 –b.” The quality of passed reads was checked and filtered using Fastp version 0.20.0 (Chen et al., 2018a). Restriction sites, base quality values < 20, and the last 5 bp of the raw reads were likely to contain errors and were thus removed to obtain clean reads with the option “–n_base_limit 5 –cut_window_size 4 –cut_mean_quality 20 –length_required 75 –qualified_quality_phred 15.” As the whole genome information of *L. oblata* was not yet reported at the time of this study, a *de novo* assemble of the GBS reference genome was generated in Stacks version 2.4 with the option “ustacks -M 3, cstacks -n 3, sstacks, tsv2bam, gstacks, populations” following Qi et al. (2018). Clean reads were aligned against the obtained GBS reference genome using Bowtie 2 version 2.3.4.1 with default parameters (Langmead and Salzberg, 2012), and subsequently genotyped and screened using GATK version 3.8–1 (McKenna et al., 2010) for SNP and INDEL predictions. Finally, the obtained SNPs were filtered using VCFtools version 0.1.16 (Danecek et al., 2011) with option “–maf 0.05 –max-missing 0.8 –minDP 4 –min-alleles 2 –max-alleles 2.”

### Genetic Diversity, Population Structure, and Phylogenetic Analysis

The number of private alleles, expected and observed heterozygosity (*H*_*E*_ and *H*_*O*_), polymorphism information content (*PIC*), nucleotide diversity (π), the effective number of alleles (*N*_*E*_), and Wright’s *F* statistics [fixation index (*F*_*ST*_) and inbreeding coefficient (*F*_*IS*_)] were calculated using the module “population” in Stacks version 2.4, VCFtools version 0.1.16, and genepop version 1.1.4 (Rousset, 2008). Reynold’s genetic distance (DR) among populations was determined from *F*_*ST*_ as follows:


DR=-ln(1-F)S⁢T.

Evolutionary clusters were identified using Admixture version 1.3.0 (Alexander et al., 2009) with the default parameters. The predefined genetic clusters (*K*) ranged between 2 and 10, each of which was repeated 10 times. The optimal value of *K* was determined using cross-validation (CV) error, which has the minimum error in Admixture. Principal component analysis (PCA) was performed by GCTA version 1.26.0 (Yang et al., 2011) to explore the genetic structure of the species. A maximum likelihood-based phylogenetic tree was performed using IQ-TREE 2 (Minh et al., 2020) to clarify the genetic relationships among populations with the coalescent SNP dataset, and node support values (ML_*BS*_) were calculated with 1,000 ultrafast bootstrap replicates.

### Demographic History Inference

The demographic history of *L. oblata* was inferred using Stairway Plot 2 (Liu and Fu, 2020). The mutation rate was set as 7.7e-9 per site per year following Pu et al. (2020). The generation time was set as seven, based on our observations from the cultivated individual since its seed stage in 2016. The tallest individual is about 0.5 m in height, and it may bloom in the spring of 2022. Though the RAD sequencing did not cover the full genome, the obtained SNP are most likely a part of the latter, which may reflect the trend of its historical demography. Two representative populations, namely, the southmost large population HDL and the small LJZ located at central were employed for demographic history inference. All samples were treated as one group, a one-dimensional site frequency spectrum (1D-SFS) was constructed using ANGSD software (Korneliussen et al., 2014), and bootstrap iterations of 200 were implemented. The result was visualized in R.

## Results

### Single-Nucleotide Polymorphisms Discovery

A total of 625, 652, 430 raw reads (91.93 Gb) were generated, resulting in 594, 664, 390 clean reads (86.82 Gb) and an average of 0.65 Gb per sample (Supplementary Table 2). The average sequencing depth of all samples was 47.33 × , with a coverage range of 87.82–96.86%. A total of 33,758 SNPs were obtained and employed for downstream analysis.

### Population Genetic Diversity and Differentiation

Table 1 reports the descriptive statistics of genetic diversity (*H*_*E*_, *H*_*O*_, *PIC*, π, and *N*_*E*_) for the seven populations of *L. oblata*. *PIC* values in all seven populations were very low (<0.19), with a value of 0.2364 at the species level. Relatively higher values of genetic diversity were observed among the four large populations (JK and JMS from YMs, WTS, and HDL from THMs) at the marginal regions compared to the three small populations located at the center (DLS, LJZ, and BJS). The northwest population WTS exhibited the highest values of *H*_*E*_ (0.2291), *H*_*O*_ (0.2054), *PIC* (0.1853), π (0.2328), and *N*_*E*_ (1.3826) (Table 1), while the lowest values of *H*_*E*_ (0.1477), *PIC* (0.1172), π (0.1625), and *N*_*E*_ (1.2563) were determined for BJS, one of the smallest populations. Furthermore, a large number of private alleles were detected in the four large populations (*N* = 1581 in JK, *N* = 256 in JMS, *N* = 724 in WTS, and *N* = 1,489 in HDL), while none were detected in the three small populations.

**TABLE 1:** The statistics of the number of samples (*N*), expected heterozygosity (*H*_*E*_), observed heterozygosity (*H*_*O*_), polymorphism information content (*PIC*), nucleotide diversity (π), efficient allelic number (*N*_*E*_), and the number of private alleles among populations (Pops).

Mountains	Pops	*N*	*H*_*E*_	*H*_*O*_	*PIC*	π	*N*_*E*_	Private alleles
Yan Mountains	JK	36	0.2015	0.1787	0.1618	0.2046	1.3421	1,581
	JMS	20	0.1928	0.1828	0.1550	0.1982	1.3270	256
Taihang Mountains	DLS	6	0.1541	0.1664	0.1232	0.1694	1.2645	0
	LJZ	8	0.1888	0.1857	0.1524	0.2027	1.3165	0
	BJS	6	0.1477	0.1932	0.1172	0.1625	1.2563	0
	WTS	34	0.2291	0.2054	0.1853	0.2328	1.3826	724
	HDL	24	0.2044	0.1794	0.1642	0.2091	1.3469	1,489
ALL		134	0.2863	0.1863	0.2364	0.2874	1.4554	4,050

A high level of genetic differentiation (*F*_*ST*_ = 0.3245) was observed at the species level, along with a low inbreeding coefficient (*F*_*IS*_ = 0.0986). The highest genetic differentiation was detected between the most northeast population JK in the YMs and the most southwest population HDL in the THMs (*F*_*ST*_ = 0.3963), while the value was minimized between the two closest (geographically) neighbored populations located at the center of its distributional range, LJZ and DLS (*F*_*ST*_ = 0.1859, Table 2). The two YM populations, JK and JMS, exhibited large genetic variations (*F*_*ST*_ = 0.3099). The southernmost population HDL differed greatly from the other THM populations (*F*_*ST*_ of 0.2626–0.3805). Notably, WTS, one of the four largest populations on the northwest side, exhibited less variation with its neighbors, namely, BJS, DLS, LJZ, and HDL, with *F*_*ST*_ values varying from 0.2320 to 0.2723 (Table 2). The higher the *F*_*ST*_ between two populations, the longer the DR (Table 2), which may also suggest a lower gene flow.

**TABLE 2:** Genetic differentiation coefficient (*F*_*ST*_) and Reynolds genetic distance (DR) among populations.

Pops	JK	JMS	LJZ	DLS	BJS	WTS	HDL
JK	–	0.3709	0.3683	0.4385	0.4974	0.3895	0.5047
JMS	0.3099	–	0.2497	0.3245	0.4460	0.3455	0.4861
LJZ	0.3081	0.2210	–	0.2057	0.3692	0.2657	0.4214
DLS	0.3550	0.2771	0.1859	–	0.4763	0.3179	0.4788
BJS	0.3919	0.3598	0.3087	0.3789	–	0.2640	0.4740
WTS	0.3226	0.2921	0.2333	0.2723	0.2320	–	0.3046
HDL	0.3963	0.3850	0.3439	0.3805	0.3775	0.2626	–

*The lower triangle presents interpopulation F_ST_, and the upper triangle presents the DR.*

### Population Structure and Phylogenetic Analysis

**FIGURE 2:**
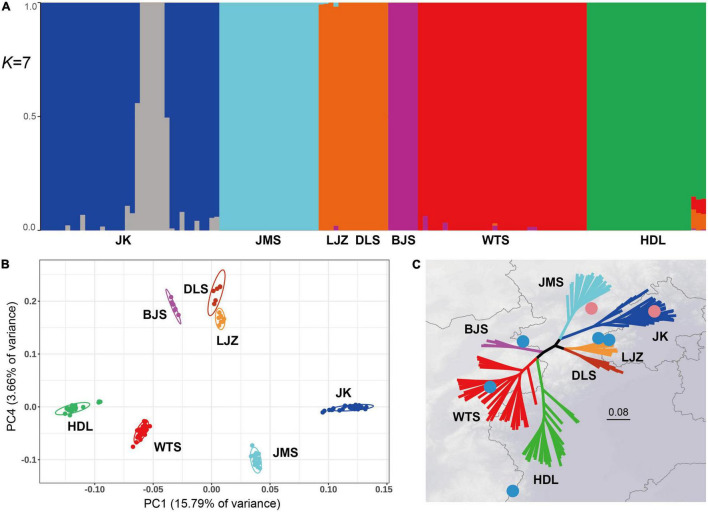
Genetic structure and phylogenetic relationships of the seven *Lonicera oblata* populations. **(A)** Admixture proportions of genetic clusters for each individual of the seven populations. The scenario of *K* = 7 is the best value according to cross-validation analysis. **(B)** Principal component analysis plot for the 134 *L. oblata* individuals based on the first two principal components. **(C)** A maximum-likelihood tree based on 33,758 single-nucleotide polymorphisms (SNPs) of the nuclear genome, with seven fully supported lineages (ML_*BS*_ = 100), which exactly correspond to species’ natural populations. The two pink circles on the northside represent populations from Yan Mountains, and the five blue circles represent populations from Taihang Mountains.

The genetic structure of *L. oblata* was investigated by Admixture (Figure 2A). The CV analysis suggested an optimal *K* value of 7 (Supplementary Figure 1). However, the seven suggested clusters did not fully correspond to their seven natural populations. Admixture suggested that LJZ and DLS formed one cluster, and it identified JK to have a fine-scale genetic structure and divided it into two clusters (Figure 2A). The first principal component (PC1) of the PCA, which explained 15.79% of all genetic variance, was able to differentiate the seven geographic groups (Figure 2B). The four large natural populations were clearly identified, and the three small populations were plotted close to each other (Figure 2B). The SNP phylogeny revealed a strong phylogeographic pattern (Figure 2C), consisting of the YM and THM clades. Seven distinct lineages were detected, corresponding exactly to the seven natural populations with full clade supports (ML_*BS*_ = 100) (Figure 2C and Supplementary Figure 2).

### Demographic History of *Lonicera oblata*

**FIGURE 3:**
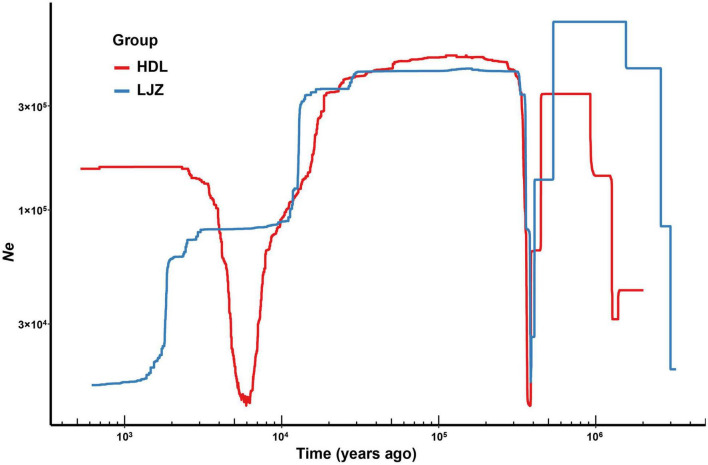
Demographic history of two populations of *Lonicera oblata*, namely, Heduling (HDL) and Lijiazhuang (LJZ) inferred by Stairway Plot 2. The x-axis indicates the time before the present, and the y-axis represents the historical effective population size.

The variations of effective population size dating from one million years ago were inferred using Stairway Plot 2 (Figure 3). The two populations of *L. oblata*, namely, HDL and LJZ, both underwent drastic decline after the Middle Pleistocene Transition (ca. 1.2–0.7 million years ago, Clark et al., 2006). Although expanded later, the population size contracted dramatically again after the Last Glacial Maximum (LGM, ca. 22 ka years ago). A recent population expansion following the LGM was suggested for HDL, while the population size of LJZ was decreasing.

## Discussion

### Genetic Composition and Variation

The genome-wide data generated by HTS are relatively easier to obtain than traditional markers, and the high number of informative loci provide us not only the solid basis for accurately characterizing the population structure (Nazareno et al., 2017) but also the exciting opportunity to address a wide range of factors for conservation genetics (Médail and Baumel, 2018; Li et al., 2020). We investigated the population genetic diversity of *L. oblata*, a critically endangered montane shrub endemic to North China, based on nuclear genomic data from the SuperGBS approach. The results reveal a low genetic diversity and strong genetic structure. *PIC* is an important index for the evaluation of genetic diversity and can be classified into three scales, such as 0 < *PIC* < 0.25, 0.25 < *PIC* < 0.5, and *PIC* > 0.5. The higher the *PIC* value, the higher the polymorphism information in the population (Botstein et al., 1980). In this study, low *PIC* (< 0.25) values were detected at the population and species level, suggesting a low degree of genetic diversity within *L. oblata*. *H*_*E*_ values determined here were markedly lower than other *Lonicera* species (e.g., *H*_*E*_ = 0.2863 in this study vs. *H*_*E*_ = 0.78 in *L. maackii* (Rupr.) Maxim.) (Barriball et al., 2015), as well as other endangered plants species [e.g., *H*_*E*_ = 0.3482 in *Tetraena mongolica* Maxim. (Cheng et al., 2020), *H*_*E*_ = 0.364 in *Firmiana danxiaensis* H. H. Hsue and H. S. Kiu (Chen et al., 2014; Table 1)]. Though only eight individuals were sampled, the LJZ population possesses a relatively high level of genetic diversity among the three small populations (JMS, DLS, and LJZ, Table 1). There are lots of mountains with altitudes of ca. 1,200 m in LJZ, whose ridges are connected. The vertical slopes between the top and the hillside in most mountains may decrease human interference and provide the opportunity for frequent genetic exchange within the population. Note that the genetic diversity of the central populations (BJS, DLS, and LJZ) was averagely lower than that of the four marginal populations (JK, JMS, WTS, and HDL) in *L. oblata* (Table 1), so the scenario of declining genetic diversity during the northward expansion of the species is not supported in this study. In contrast to the low genetic diversity, we detected pronounced genetic structure within *L. oblata* (*F*_*ST*_ = 0.3245), implying a long history of independent evolution and less gene flow among populations.

The population genetic composition is generally a function of both the species-specific biological characteristics and the corresponding ecological factors. The low genetic diversity and high genetic differentiation detected for *L. oblata* may be attributed to the following factors. (i) The extremely low numbers both at the population and individual level. The only seven uneven populations are highly fragmented and scattered at the top of mountains in a broad geographic range, and the limestone-preferred habitat of the species has been disturbed by long-term anthropogenic activities throughout history. The strong genetic differentiation among populations of *L. oblata* implies that the phenomenon of sky islands, which is significant in southwest China, may also occur in the THMs and the YMs in North China. (ii) The combined side effect of its reproductive system and fruit dispersal characteristics. A mixed mating system of biased outcross and partial self-fertilization is observed in *L. oblata*, and insects are necessary for pollination (Wu et al., 2022), while pollen limitation occurs during its flowering period. The harsh climate conditions during its flowering period (strong wind, sudden drop in temperature, snow, and/or dusty wind in the early spring) greatly interfere with the activities of effective pollinators and the pollination success of *L. oblata*. The highly fragmented populations and persistent strong winds at the top of hills and cliffs further inhibit pollination. The red fleshy fruits of *L. oblata* are attractive to birds, thus providing the possibility of long-distance dispersal of seeds and genetic exchange among populations. However, only a small-bodied bird *Zosterops* was recorded to feed on the fruits of *L. oblata* during our field investigation in Beijing in July. *Zosterops* live in a distance of ca. < 2 km in thick forests during the breeding season (May to July). Extending the distributional range of *L. oblata* over the contribution of birds like *Zosterops* is difficult. (iii) Seedlings barely survive in the field, which further decreases the genetic exchange between populations. Seedlings were difficult to find in the community during our field investigations, and an obvious inverted triangle population pyramid was observed (unpublished data). Hence, multiple negative effects may contribute to the strong genetic structure of *L. oblata*, whose destiny may be further challenged by future climate changes (Wu et al., 2021).

### Population Structure and Glacial Refugia

The genetic structure of a population provides further information on the evolution of a species. In this study, a significant phylogeographic structure was identified from the structure, principal component, and phylogeny analyses, suggesting the division of seven groups (Figure 2). However, the seven clusters identified in Admixture did not correspond to those in the latter two analyses. In particular, the Admixture analysis placed DLS and LJZ into a single cluster, while the eastmost large population JK was divided into two clusters (Figure 2A). When *K* = 5, samples of JK did not differentiate intrapopulations, and the three small populations, namely, BJS, DLS, and LJZ, compose one cluster (Supplementary Figure 3). Such a scenario suggests an Admixture identity of these three small populations distributed in the species’ central region. The presence of two clusters in JK under the scenario *K* = 7 is difficult to explain. The six samples from one of the JK clusters (JK20–JK25) were randomly selected in the field just like the other samples. There were no distinct characteristics (e.g., elevation, slope direction, and canopy density) identified for the six samples compared to the remaining samples. JK is strongly exposed to tourism activity compared to the other six populations. The Jiankou Great Wall, a grand section of the Great Wall, attracts thousands of tourists each year. Many *L. oblata* individuals grow right on the Great Wall and are threatened by tourists during their climb. Some deaths of individuals due to the direct damage from tourists were witnessed during our fieldwork. Future work will include fine-scale sampling and landscape genomic study in order to understand the fine-scale genetic structure within JK.

The PCA and phylogenetic analysis revealed the seven identified groups to fully correspond to the natural populations of the species. Clade support values do not generally reach high levels in the study of population genetic diversity, and the sample(s) of one population may sometimes be resolved in the lineage of another population, such as cushion willow (Chen et al., 2019), grape (Liang et al., 2019), *Prunus* (Liang et al., 2019), and *Q. aquifolioides* Rehder and E. H. Wilson (Du et al., 2017). In this study, we obtained a well-resolved phylogeny of *L. oblata* at the population level. The phylogenetic tree consisted of two key clades, namely, the THMs and YMs, and the seven natural populations were all resolved as monophyly with full clade supports on the molecular tree (ML_*BS*_ = 100) (Figure 2C). Both the strong phylogeographic pattern and the fully supported clade values at the population level suggest the seven natural populations to be evolutionary significant units following the definition of Moritz (1994). Strong phylogeographic characteristics combined with great genetic variation among populations (Table 2) also imply these populations be defined as management units following Avise (2000). Among them, the four large populations (JK and JMS in the YMs and WTS and HDL in the THMs), which have plenty of private alleles, are of greater importance as primary conservation units. The high level of genetic differentiation and well-resolved phylogeny in *L. oblata* may also suggest that long-term vicariance exists among these highly fragmented populations, as well as a low gene flow.

The NNE trending THMs and EW trending YMs act as dispersal corridors not only between the northeast cool-temperate and southern warm-temperate forests but also among fragmented populations of endangered species in China. These two great mountains in North China may also provide potential refugia for species during the global climate changes of the Quaternary (Zeng et al., 2011; Hou et al., 2020; Lin et al., 2021). Our results demonstrate many of the extant populations of *L. oblata* are genetically isolated. A high level of genetic diversity and plentiful private alleles were detected in the four large populations located in the marginal region of the *L. oblata* distribution range (*N* = 1,581 in JK, *N* = 256 in JMS, *N* = 724 in WTS, and *N* = 1,489 in HDL, Table 1). In contrast, low genetic diversity and no private alleles were detected in the populations located in the central region (LJZ, DLS, and BJS). Our ecological niche modeling analysis (ENM) of *L. oblata* suggests a wide historical distribution range, covering its current range since the LGM, followed by a dramatic decline to the present (Wu et al., 2021). Affected by the intensive uplift of both the Qinghai-Tibetan Plateau during the Late Cenozoic (Li et al., 2001) and THMs and YMs during the Late Pliocene to Pleistocene (Wu et al., 1999), which intensified monsoon while enhanced aridity in the Asian interior and repeated glacial events during the Quaternary, historical climate and geographic characteristics of northern China varied greatly. The demographic history of *L. oblata* may also have suffered from these factors, particularly the LGM, and its population size decreased sharply (Figure 3). This might also explain that no private allele was detected in the three small current populations, namely, BJS, DLS, and LJZ. Taken together, the genomic data and ENM suggest the existence of LGM refugia for *L. oblata* in the THMs and YMs, and the hypothesis of *in situ* survival is supported. The two greatest mountains in North China, namely the THMs and the YMs, may have acted as “Noah’s Ark” for numerous plant lineages during the LGM, providing vital refugia for the postglacial preservation of biodiversity in North China.

### Threats and Conservation Suggestions for *Lonicera oblata*

The endangerment of species may be a result of both internal and/or external factors. Our field investigation suggests the possible worsening of the *L. oblata* endangerment situation. Although it occupies a relatively large distribution range in North China, only seven populations and a total of ca. 1,000 individuals of the species were recorded. Its limestone-specific habitat may greatly restrict its expansion, and the limited populations are highly fragmented and affected by human activities (e.g., agriculture, tourism, and logging). Furthermore, pollen limitation and harsh climate conditions during its flowering period restrain its survival and reproduction (Wu et al., 2022). Our results reveal a low genetic diversity and high genetic differentiation, which may suggest a reduction in the species fitness. Moreover, potentially suitable regions for the expansion of the species are limited (Wu et al., 2021). The dry Chinese Loess Plateau to the west of the THMs, the cold and dry desert in the Yin Mountains to the north of the YMs, and the cold temperature in Northeast China all constrain the future expansion of *L. oblata* severely. Due to the great conservation achievements of forestry and ecology across China in recent decades, the majority of previously bare mountaintops are now densely occupied by woody plants. This may also act as a challenge to the expansion of *L. oblata*, which favors an open habitat. Thus, this critically endangered species endemic to North China is currently facing the plight of “nowhere to go” (Nogués-Bravo et al., 2007; Loarie et al., 2009).

The exploration of population genetic patterns can provide vital information for the conservation and management of threatened species. Considering the aforementioned threatening factors, several conservation suggestions are proposed here: (i) forceful *in situ* conservation should be provided at both the population and the individual level; (ii) JK, JMS, HDL, and WTS populations should be given priority among the seven conservation units, including the construction of mini-reserves for the former three that are not distributed in national parks or nature reserves, and forceful conservation actions should be performed; (iii) the collection and long-term preservation of seeds from multi-populations should be accomplished, with the timely determination of seed germination and artificial propagation techniques; and (iv) as the intersection regions between the north of the THMs and west of the YMs are predicted to provide stable climate conditions in the future (Wu et al., 2021), this area should be given the priority to experiments of reintroduction and *ex situ* conservation.

## Conclusion

In this study, we performed a genome-wide population genetic investigation on *L. oblata*, a highly threatened montane shrub endemic to North China, whose natural distribution range lies in the contact zone between southern warm-temperate and northern cold-temperate forests. Based on the SuperGBS method, we determined a low level of genetic diversity, a high degree of genetic differentiation, and a strong phylogeographic structure. Sharp population size contraction was suggested, which occurred after the middle Pleistocene Transition and the LGM, respectively. The results from the current genomic data combined with our previous ENM analysis suggest the existence of LGM refugia in the THMs and YMs. Affected by multiple factors relating to geography, climate, and biological and ecological characteristics, the future of *L. oblata* is challenged by the “nowhere to go” scenario. Four key conservation units were identified for *L. oblata*, and several conservation suggestions were provided. Further work characterizing the whole genome of *L. oblata* and a fine-scale landscape genomic study should be performed especially on the JK population with the aim to understand how geographic and ecological factors shape its genetic structure and evolutionary history. Our population genomic study provides valuable information for the conservation and management of *L. oblata* and contributes to the further understanding of the role of the THMs and YMs in preserving the biodiversity in North China.

## Data Availability Statement

The datasets presented in this study can be found in online repositories. The names of the repository/repositories and accession number(s) can be found below: https://www.ncbi.nlm.nih.gov/genbank/, PRJNA787048.

## Author Contributions

X-YM conceptualized, supervised the study, analyzed the data, wrote, and revised the draft. Y-MW, X-LS, LT, F-WL, X-FX, and YN contributed to the fieldwork and collected the materials. Y-MW contributed to the species distribution maps. All authors contributed to the article and approved the submitted version.

## Conflict of Interest

The authors declare that the research was conducted in the absence of any commercial or financial relationships that could be construed as a potential conflict of interest.

## Publisher’s Note

All claims expressed in this article are solely those of the authors and do not necessarily represent those of their affiliated organizations, or those of the publisher, the editors and the reviewers. Any product that may be evaluated in this article, or claim that may be made by its manufacturer, is not guaranteed or endorsed by the publisher.

## Footnotes

1
http://www.forestry.gov.cn/main/5461/20210908/162515850572900.html

## Supplementary Material

Supplementary Table S1 Population information.

Supplementary Table S2 Sequencing information.

Supplementary Figure S1 The optimal *K* value.

Supplementary Figure S2 Phylogenetic tree of 134 individuals based on SNP data matrix.

Supplementary Figure S3 Structure analysis of *K* = 2–10.

## References

[B1] Alexander D. H. Novembre J. Lange K. (2009). Fast model-based estimation of ancestry in unrelated individuals. *Genome Res.* 19 1655–1664. 10.1101/gr.094052.109 19648217 PMC2752134

[B2] Avise J. C. (2000). *Phylogeography — the History and Formation of Species.* Cambridge, MA: Harvard University Press.

[B3] Bai W. L. Liao W. J. Zhang D. Y. (2010). Nuclear and chloroplast DNA phylogeography reveal two refuge areas with asymmetrical gene flow in a temperate walnut tree from East Asia. *New Phytol.* 188 892–901. 10.1111/j.1469-8137.2010.03407.x 20723077

[B4] Barriball K. McNutt E. J. Gorchov D. L. Rocha O. J. (2015). Inferring invasion patterns of *Lonicera maackii* (Rupr) Herder (Caprifoliaceae) from the genetic structure of 41 naturalized populations in a recently invaded area. *Biol. Invasions* 17 2387–2402. 10.1007/s10530-015-0882-7

[B5] Botstein D. White R. L. Skolnick M. Davis R. W. (1980). Construction of a genetic linkage map in man using restriction fragment length polymorphisms. *Am. J. Hum. Genet.* 32 314–331. 6247908 PMC1686077

[B6] Boukteb A. Sakaguchi S. Ichihashi Y. Kharrat M. Nagano A. J. Shirasu K. (2021). Analysis of genetic diversity and population structure of *Orobanche foetida* populations from *Tunisia* using RADseq. *Front. Plant Sci.* 12:618245. 10.3389/fpls.2021.618245 33927733 PMC8078179

[B7] Cardinale B. J. Duffy J. E. Gonzalez A. Hooper D. U. Perrings C. Venail P. (2012). Biodiversity loss and its impact on humanity. *Nature* 486 59–67. 10.1038/nature11148 22678280

[B8] Catchen J. Hohenlohe P. A. Bassham S. Amores A. Cresko W. A. (2013). Stacks: an analysis tool set for population genomics. *Mol. Ecol.* 22 3124–3140. 10.1111/mec.12354 23701397 PMC3936987

[B9] Cavender N. Westwood M. Bechtoldt C. Donnelly G. Oldfield S. Gardner M. (2015). Strengthening the conservation value of ex situ tree collections. *Oryx* 49 416–424. 10.1017/S0030605314000866

[B10] Chapin F. S. III Zavaleta E. S. Eviner V. T. Naylor R. L. Vitousek P. M. Reynolds H. L. (2000). Consequences of changing biodiversity. *Nature* 405 234–242. 10.1038/35012241 10821284

[B11] Charlesworth D. Willis J. (2009). The genetics of inbreeding depression. *Nat. Rev. Genet.* 10 783–796. 10.1038/nrg2664 19834483

[B12] Chen J. H. Huang Y. Brachi B. Yun Q. Z. Zhang W. Lu W. (2019). Genome-wide analysis of Cushion willow provides insights into alpine plant divergence in a biodiversity hotspot. *Nat. Commun.* 10:5230. 10.1038/s41467-019-13128-y 31745089 PMC6864086

[B13] Chen S. Li M. Hou R. Liao W. Zhou R. Fan Q. (2014). Low genetic diversity and weak population differentiation in *Firmiana danxiaensis*, a tree species endemic to Danxia landform in northern Guangdong, China. *Biochem. Syst. Ecol.* 55 66–72. 10.1016/j.bse.2014.02.029

[B14] Chen Y. S. Deng T. Zhou Z. Sun H. (2018b). Is the East Asian flora ancient or not? *Natl. Sci. Rev.* 5 920–932. 10.1093/nsr/nwx156

[B15] Chen S. Zhou Y. Chen Y. Gu J. (2018a). Fastp: an ultra-fast all-in-one FASTQ preprocessor. *Bioinformatics* 34 i884–i890. 10.1093/bioinformatics/bty560 30423086 PMC6129281

[B16] Cheng J. Kao H. X. Dong S. B. (2020). Population genetic structure and gene flow of rare and endangered *Tetraena mongolica* Maxim. revealed by reduced representation sequencing. *BMC Plant Biol.* 20:391. 10.1186/s12870-020-02594-y 32842966 PMC7448513

[B17] Clark P. U. Archer D. Pollard D. Blum J. D. Rial J. A. Brovkin V. (2006). The middle Pleistocene transition: characteristics, mechanisms, and implications for long-term changes in atmospheric pCO2. *Quat. Sci. Rev.* 25 3150–3184. 10.1016/j.quascirev.2006.07.008

[B18] Cole C. T. (2003). Genetic variation in rare and common plants. *Ann. Rev. Ecol. Evol. Syst.* 34 213–237. 10.1146/annurev.ecolsys.34.030102.151717

[B19] Danecek P. Auton A. Abecasis G. Albers C. A. Banks E. DePristo M. A. (2011). The variant call format and VCFtools. *Bioinformatics* 27 2156–2158. 10.1093/bioinformatics/btr330 21653522 PMC3137218

[B20] Davey J. W. Hohenlohe P. A. Etter P. D. Boone J. Q. Catchen J. M. Blaxter M. L. (2011). Genome-wide genetic marker discovery and genotyping using next-generation sequencing. *Nat. Rev. Genet.* 12 499–510. 10.1038/nrg3012 21681211

[B21] Du F. K. Hou M. Wang W. Mao K. Hampe A. (2017). Phylogeography of *Quercus aquifolioides* provides novel insights into the Neogene history of a major global hotspot of plant diversity in south-west China. *J. Biogeogr.* 44 294–307. 10.1111/jbi.12836

[B22] Feng J. Zhao S. Li M. Zhang C. Qu H. Li Q. (2020). Genome-wide genetic diversity detection and population structure analysis in sweetpotato (*Ipomoea batatas*) using RAD-seq. *Genomics* 112 1978–1987. 10.1016/j.ygeno.2019.11.010 31756427

[B23] Glover K. A. Hansen M. M. Lien S. Als T. D. Høyheim B. Skaala Ø (2010). A comparison of SNP and STR loci for delineating population structure and performing individual genetic assignment. *BMC Genet.* 11:2. 10.1186/1471-2156-11-2 20051144 PMC2818610

[B24] Guo X. D. Wang H. F. Bao L. Wang T. M. Bai W. L. Ye J. W. (2014). Evolutionary history of a widespread tree species Acer mono in East Asia. *Ecol. Evol.* 4 4332–4345. 10.1002/ece3.1278 25540694 PMC4267871

[B25] Guo Z. T. Sun B. Zhang Z. S. Peng S. Z. Xiao G. Q. Ge J. Y. (2008). A major reorganization of Asian climate by the early Miocene. *Clim. Past* 4 153–174. 10.5194/cp-4-153-2008

[B26] He K. Jiang X. L. (2014). Sky islands of southwest China. I. An overview of phylogeographic patterns. *Chin. Sci. Bull.* 59 585–597. 10.1007/s11434-013-0089-1

[B27] Hou H. Ye H. Wang Z. Wu J. Gao Y. Han W. (2020). Demographic history and genetic differentiation of an endemic and endangered *Ulmus lamellosa* (Ulmus). *BMC Plant Biol.* 20:526. 10.1186/s12870-020-02723-7 33203402 PMC7672979

[B28] Işik K. Kani (2011). Rare and endemic species: why are they prone to extinction? *Turk. J. Bot.* 35 411–417. 10.3906/bot-1012-90 31411186

[B29] Korneliussen T. S. Albrechtsen A. Nielsen R. (2014). ANGSD: analysis of next generation sequencing data. *BMC Bioinformatics* 15:356. 10.1186/s12859-014-0356-4 25420514 PMC4248462

[B30] Langmead B. Salzberg S. L. (2012). Fast gapped-read alignment with Bowtie 2. *Nat. Methods* 9 357–359. 10.1038/nmeth.1923 22388286 PMC3322381

[B31] Li J. Milne R. I. Ru D. Miao J. Tao W. Zhang L. (2020). Allopatric divergence and hybridization within *Cupressus chengiana* (Cupressaceae), a threatened conifer in the northern Hengduan Mountains of western China. *Mol. Ecol.* 29 1250–1266. 10.1111/mec.15407 32150782

[B32] Li J. J. Fang X. M. Pan B. T. Zhao Z. J. Song Y. G. (2001). Late Cenozoic intensive uplift of Qinghai-Xizang Plateau and its impacts on environments in surrounding area. *Quat. Sci.* 21 381–391. 10.3321/j.issn:1001-7410.2001.05.001 30704229

[B33] Liang Z. Duan S. Sheng J. Zhu S. Ni X. Shao J. (2019). Whole-genome resequencing of 472 Vitis accessions for grapevine diversity and demographic history analyses. *Nat. Commun.* 10:1190. 10.1038/s41467-019-09135-8 30867414 PMC6416300

[B34] Lin N. Landis J. B. Sun Y. X. Huang X. H. Zhang X. Liu Q. (2021). Demographic history and local adaptation of *Myripnois dioica* (Asteraceae) provide insight on plant evolution in northern China flora. *Ecol. Evol.* 11 8000–8013. 10.1002/ece3.7628 34188867 PMC8216978

[B35] Liu C. Tsuda Y. Shen H. Hu L. Saito Y. Ide Y. (2014). Genetic structure and hierarchical population divergence history of Acer mono var. mono in south and northeast China. *PLoS One* 9:e87187. 10.1371/journal.pone.0087187 24498039 PMC3909053

[B36] Liu X. M. Fu Y. X. (2020). Stairway Plot 2: demographic history inference with folded SNP frequency spectra. *Genome Biol.* 21:280. 10.1186/s13059-020-02196-9 33203475 PMC7670622

[B37] Loarie S. R. Duffy P. B. Hamilton H. Asner G. P. Field C. B. Ackerly D. D. (2009). The velocity of climate change. *Nature* 462 1052–1055. 10.1038/nature08649 20033047

[B38] Lu L. M. Mao L. F. Yang T. Ye J. F. Liu B. Li H. L. (2018). Evolutionary history of the angiosperm flora of China. *Nature* 554 234–238. 10.1038/nature25485 29420476

[B39] Luo Z. Brock J. Dyer J. M. Kutchan T. Schachtman D. Augustin M. (2019). Genetic diversity and population structure of a *Camelina sativa* spring panel. *Front. Plant Sci.* 10:184. 10.3389/fpls.2019.00184 30842785 PMC6391347

[B40] McKenna A. Hanna M. Banks E. Sivachenko A. Cibulskis K. Kernytsky A. (2010). The genome analysis Toolkit: a MapReduce framework for analyzing next-generation DNA sequencing data. *Genome Res.* 20 1297–1303. 10.1101/gr.107524.110 20644199 PMC2928508

[B41] Médail F. Baumel A. (2018). Using phylogeography to define conservation priorities: the case of narrow endemic plants in the Mediterranean Basin hotspot. *Biol. Conserv.* 224 258–266. 10.1016/j.biocon.2018.05.028

[B42] Minh B. Q. Schmidt H. A. Chernomor O. Schrempf D. Woodhams M. D. von Haeseler A. (2020). IQ-TREE 2: new models and efficient methods for phylogenetic inference in the genomic era. *Mol. Biol. Evol.* 37 1530–1534. 10.1093/molbev/msaa015 32011700 PMC7182206

[B43] Moritz C. (1994). Applications of mitochondrial DNA analysis in conservation: a critical review. *Mol. Ecol.* 3 401–411. 10.1111/j.1365-294X.1994.tb00080.x

[B44] Myers N. Mittermeier R. A. Mittermeier C. G. Da Fonseca G. A. B. Kent J. (2000). Biodiversity hotspots for conservation priorities. *Nature* 403 853–858. 10.1038/35002501 10706275

[B45] Nazareno A. G. Bemmels J. B. Dick C. Lohmann L. G. (2017). Minimum sample sizes for population genomics: an empirical study from an Amazonian plant species. *Mol. Ecol.* 17 1136–1147. 10.1111/1755-0998.12654 28078808

[B46] Nogués-Bravo D. Araújo M. B. Errea M. P. Martínez-Rica J. P. (2007). Exposure of global mountain systems to climate warming during the 21st Century. *Glob. Environ. Chang.* 17 420–428. 10.1016/j.gloenvcha.2006.11.007

[B47] Pu X. D. Li Z. Tian Y. Gao R. R. Hao L. J. Hu Y. T. (2020). The honeysuckle genome provides insight into the molecular mechanism of carotenoid metabolism underlying dynamic flower coloration. *New Phytol.* 227:930e43. 10.1111/nph.16552 32187685 PMC7116227

[B48] Puritz J. B. Matz M. V. Toonen R. J. Weber J. N. Bolnick D. I. Bird C. E. (2014). Demystifying the RAD fad. *Mol. Ecol.* 23 5937–5942. 10.1111/mec.12965 25319241

[B49] Qi P. Gimode D. Saha D. Schröder S. Chakraborty D. Wang X. (2018). UGbS-Flex, a novel bioinformatics pipeline for imputation-free SNP discovery in polyploids without a reference genome: finger millet as a case study. *BMC Plant Biol.* 18:117. 10.1186/s12870-018-1316-3 29902967 PMC6003085

[B50] Qiao Y. Guo F. Huo N. Zhan L. Sun J. Zuo X. (2021). Genotyping-by-sequencing to determine the genetic structure of a Tibetan medicinal plant *Swertia mussotii* Franch. *Genet. Resour. Crop Evol.* 68 469–484. 10.1007/s10722-020-00993-6

[B51] Qiu Y. Fu C. Comes H. P. (2011). Plant molecular phylogeography in China and adjacent regions: tracing the genetic imprints of Quaternary climate and environmental change in the world’s most diverse temperate flora. *Mol. Phylogenet. Evol.* 59 225–244. 10.1016/j.ympev.2011.01.012 21292014

[B52] Rousset F. (2008). Genepop’ 007: a complete re-implementation of the genepop software for Windows and Linux. *Mol. Ecol. Resour.* 8 103–106. 10.1111/j.1471-8286.2007.01931.x 21585727

[B53] Sonah H. Bastien M. Iquira E. Tardivel A. Légaré G. Boyle B. (2013). An improved genotyping by sequencing (GBS) approach offering increased versatility and efficiency of SNP discovery and genotyping. *PLos One* 8:e54603. 10.1371/journal.pone.0054603 23372741 PMC3553054

[B54] Wang H. Li J. M. Wu T. W. (2018). Characteristics and genesis of geoheritage resources of Taihang Mountain. *Acta Sci. Nat. Univ. Pekin.* 54 546–554. 10.13209/j.0479-8023.2017.098

[B55] Wang H. S. Zhang Y. L. Huang J. S. Wu Z. F. Zhao S. L. Wang H. S. (1995). A floristic study on the seed plants in the North China region. *Acta Bot. Yunnanica* 7 32–54.

[B56] Wang N. Thomson M. Bodles W. J. A. Crawford R. M. M. Hunt H. V. Featherstone A. W. (2013). Genome sequence of dwarf birch (*Betula nana*) and cross-species RAD markers. *Mol. Ecol.* 22 3098–3111. 10.1111/mec.12131 23167599

[B57] Wang S. H. Bao L. Wang T. M. Wang H. F. Ge J. P. (2016). Contrasting genetic patterns between two coexisting *Eleutherococcus* species in northern China. *Ecol. Evol.* 6 3311–3324. 10.1002/ece3.2118 27103988 PMC4833501

[B58] Wang Y. Li H. (2008). Initial formation and Mesozoic tectonic exhumation of an intracontinental tectonic belt of the northern part of the Taihang Mountain Belt, Eastern Asia. *J. Geol.* 116 155–172. 10.1086/529153

[B59] Wu C. Zhang X. Ma Y. (1999). The Taihang and Yan mountains rose mainly in Quaternary. *North China Earthquake Sci.* 17 1–7. 10.1007/s11103-011-9753-5 21327834

[B60] Wu Y. M. Shen X. L. Tong L. Lei F. W. Mu X. Y. Zhang Z. X. (2021). Impact of past and future climate change on the potential distribution of an endangered montane shrub *Lonicera oblata* and its conservation implications. *Forests* 12:125. 10.3390/f12020125

[B61] Wu Y. M. Shen X. L. Tong L. Lei F. W. Xia X. F. Mu X. Y. (2022). Reproductive biology of an endangered lithophytic shrub and implications for its conservation. *BMC Plant Biol.* 22:80. 10.1186/s12870-022-03466-3 35193519 PMC8862588

[B62] Xiong S. Zhao Y. Chen Y. Gao M. Wu L. Wang Y. (2020). Genetic diversity and population structure of *Quercus fabri* Hance in China revealed by genotyping-by-sequencing. *Ecol. Evol.* 10 8949–8958. 10.1002/ece3.6598 32884670 PMC7452768

[B63] Yang J. Lee S. H. Goddard M. E. Visscher P. M. (2011). GCTA: a tool for genome-wide complex trait analysis. *Am. J. Hum. Genet.* 88 76–82. 10.1016/j.ajhg.2010.11.011 21167468 PMC3014363

[B64] Ye J. W. Yuan Y. G. Cai L. Wang X. J. (2017). Research progress of phylogeographic studies of plant species in temperate coniferous and broadleaf mixed forests in Northeastern China. *Biodivers. Sci.* 25 1339–1349. 10.17520/biods.2017265 34063014

[B65] Ye J. W. Zhang Z. K. Wang H. F. Bao L. Ge J. P. (2019). Phylogeography of *Schisandra chinensis* (Magnoliaceae) reveal multiple refugia with ample gene flow in northeast China. *Front. Plant Sci.* 10:199. 10.3389/fpls.2019.00199 30858859 PMC6397880

[B66] Zeng Y. F. Liao W. J. Petit R. J. Zhang D. Y. (2011). Geographic variation in the structure of oak hybrid zones provides insights into the dynamics of speciation. *Mol. Ecol.* 20 4995–5011. 10.1111/j.1365-294X.2011.05354.x 22059561

[B67] Zeng Y. F. Wang W. T. Liao W. J. Wang H. F. Zhang D. Y. (2015). Multiple glacial refugia for cool-temperate deciduous trees in northern East Asia: the Mongolian Oak as a case study. *Mol. Ecol.* 24 5676–5691. 10.1111/mec.13408 26439083

[B68] Zhu Y. X. Wu Y. M. Shen X. L. Tong L. Xia X. F. Mu X. Y. (2019). The complete chloroplast genome of *Lonicera oblata*, a critically endangered species endemic to North China. *Mitochondrial DNA B* 4 2337–2338. 10.1080/23802359.2019.1629344 33365532 PMC7687385

