# Supplementary material for: Genomic Data Reveals Profound Genetic Structure and Multiple Glacial Refugia in Lonicera oblata (Caprifoliaceae), a Threatened Montane Shrub Endemic to North China
Source: Front Plant Sci. 2022 May 9;13:832559. doi: 10.3389/fpls.2022.832559 (PMC9125190; doi:10.3389/fpls.2022.832559)
Supplement: Supplementary Table S1 — Population information. [file Table_1.DOCX]

Table S1 Population information of *Lonicera oblata*.

| Population | Longitude | Latitude | Elevation (m) | Location |
| --- | --- | --- | --- | --- |
| JK | 116.4943 | 40.4638 | 971 | Jiankou Great Wall, Beijing, China |
| JMS | 115.2984 | 40.4723 | 1086 | Jiming Mountian, Hebei, China |
| DLS | 115.4643 | 39.9795 | 1387 | Dongling Mountian, Beijing, China |
| LJZ | 115.5977 | 39.9813 | 1239 | Lijiazhuang Village, Beijing, China |
| BJS | 114.1561 | 39.9140 | 1149 | Bijia Mountian, Hebei, China |
| WTS | 113.6251 | 39.1836 | 1967 | Wutai Mountian, Shanxi, China |
| HDL | 113.9730 | 37.4222 | 1528 | Heduling Great Wall, Hebei, China |
