# Supplementary figures and images for: Genomic Data Reveals Profound Genetic Structure and Multiple Glacial Refugia in Lonicera oblata (Caprifoliaceae), a Threatened Montane Shrub Endemic to North China
Source: Front Plant Sci. 2022 May 9;13:832559. doi: 10.3389/fpls.2022.832559 (PMC9125190; doi:10.3389/fpls.2022.832559)

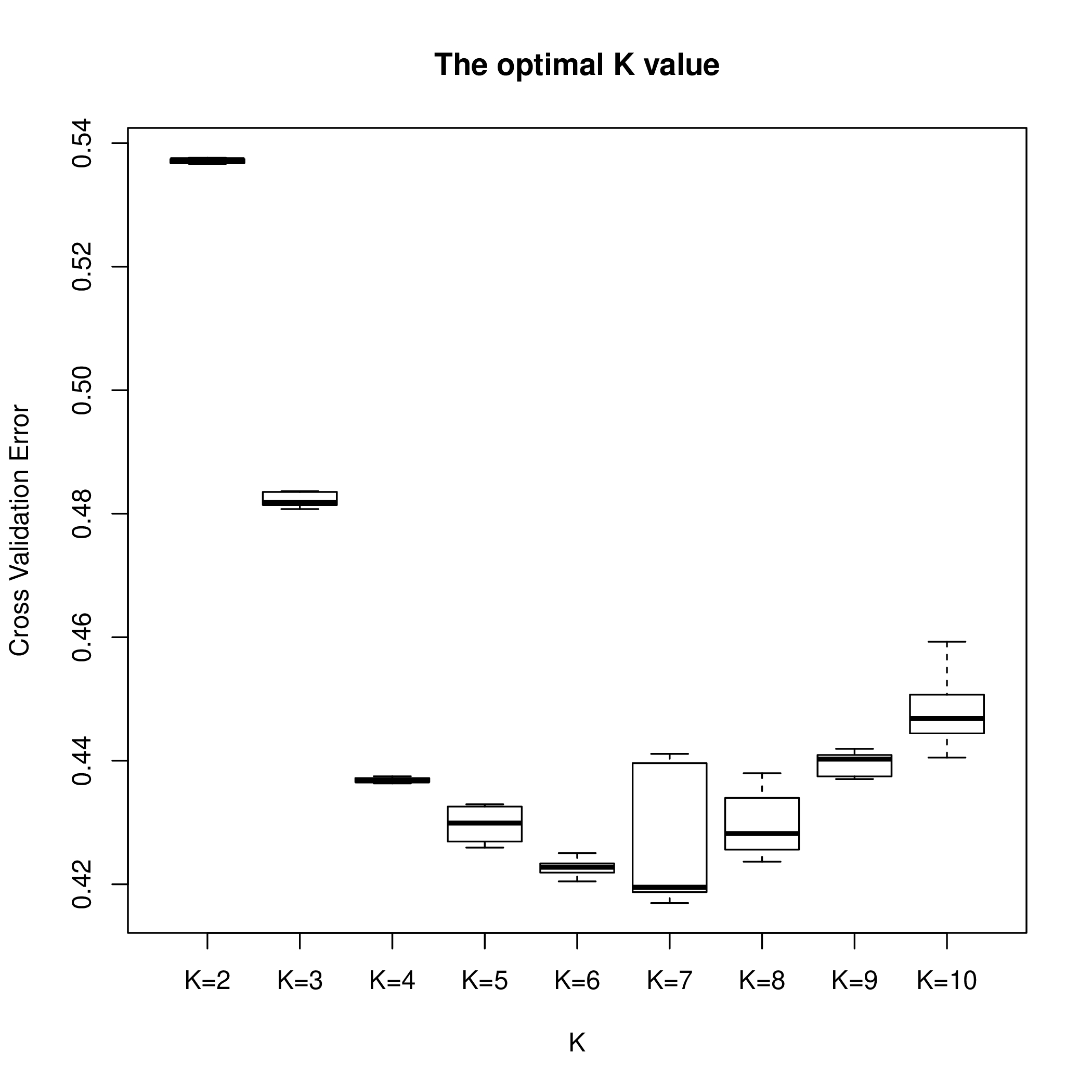

Supplement: Supplementary Figure S1 — The optimal K value. [file Image_1.PNG]

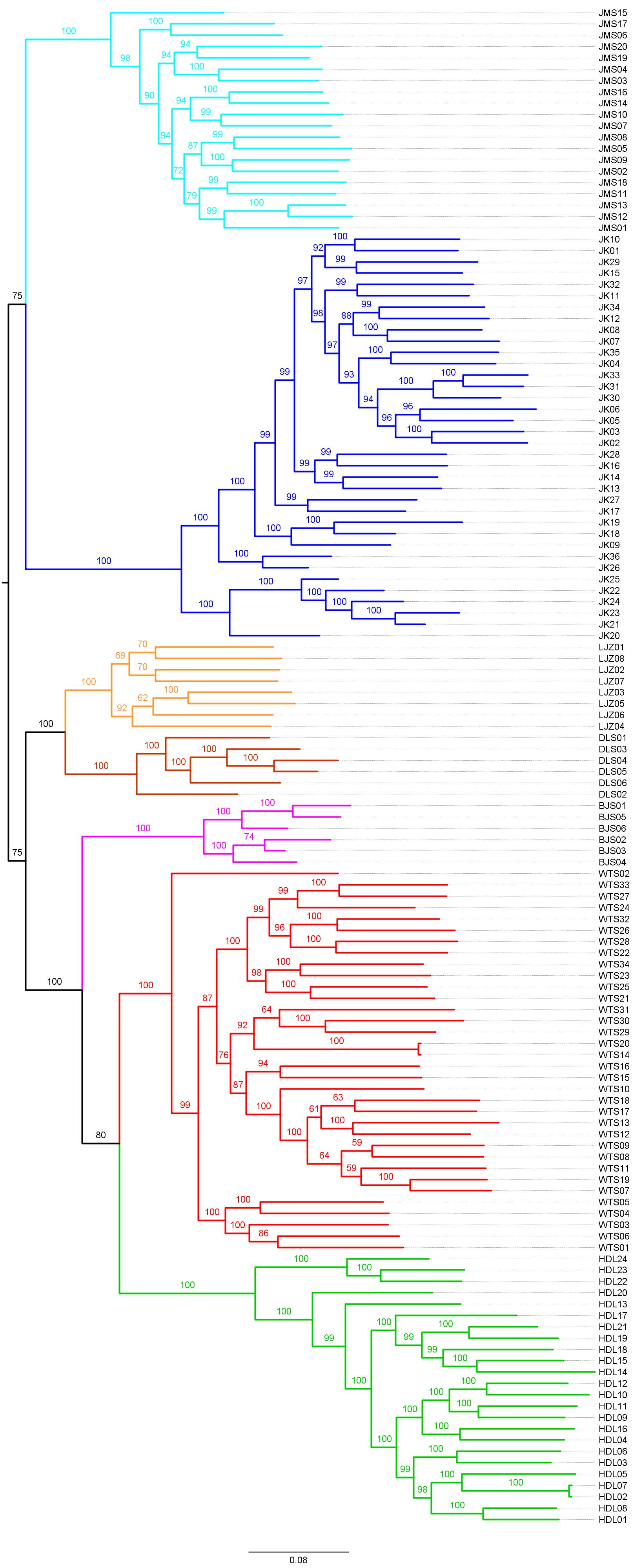

Supplement: Supplementary Figure S2 — Phylogenetic tree of 134 individuals based on SNP data matrix. [file Image_2.JPEG]

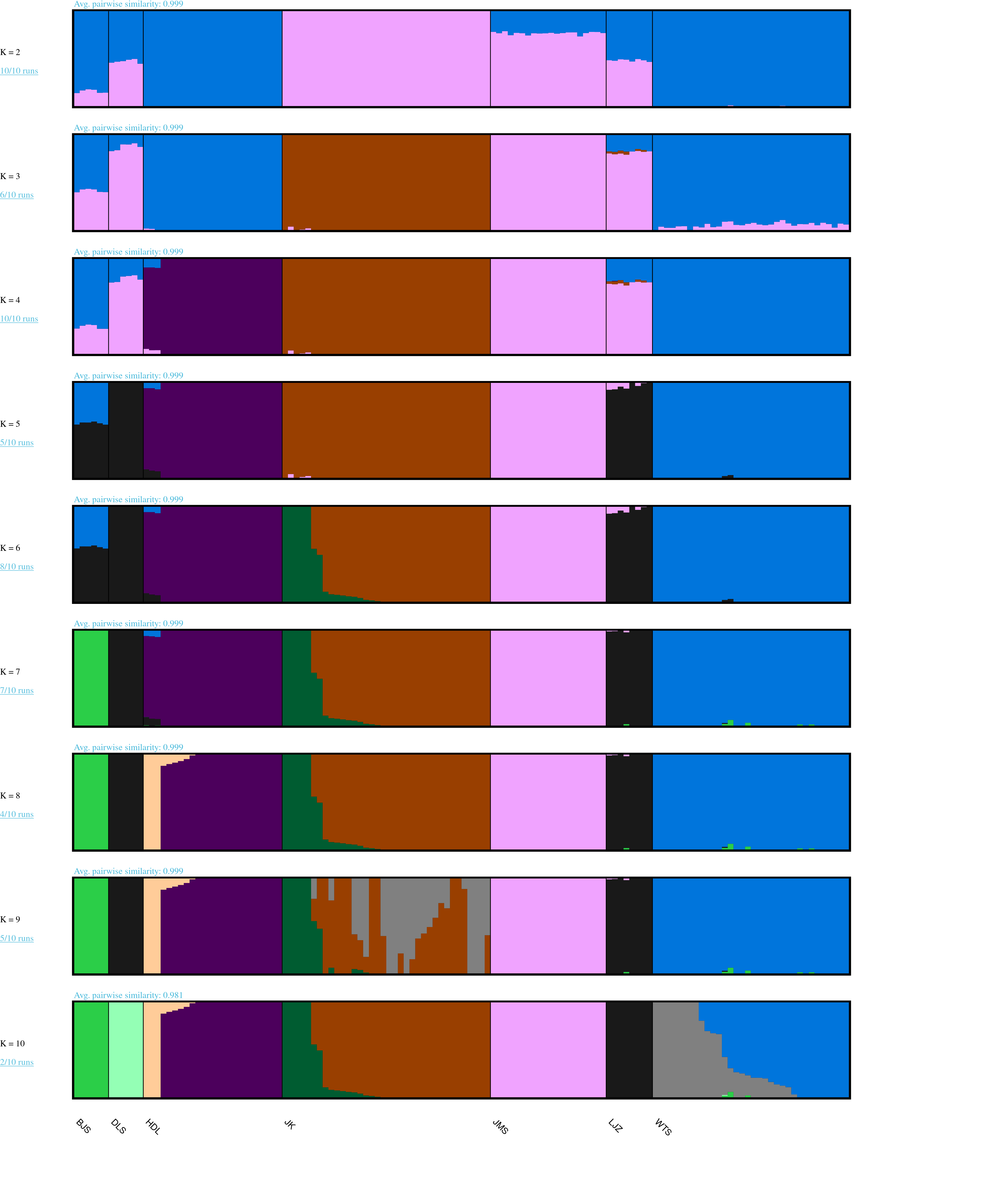

Supplement: Supplementary Figure S3 — Structure analysis of K = 2–10. [file Image_3.PNG]
